# Antifungal Effects and Mechanism of Dihydrochelerythrine Against *Fusarium oxysporum*

**DOI:** 10.3390/microorganisms13122800

**Published:** 2025-12-09

**Authors:** Hongshuai Yang, Zixue Wang, Zhiyuan Guan, Min Zhao, Hao Wu, Hongyan Yang

**Affiliations:** 1 College of Life Sciences, Northeast Forestry University, Harbin 150040, China; 2 Key Laboratory for Enzyme and Enzyme-like Material Engineering of Heilongjiang, Harbin 150040, China

**Keywords:** *Fusarium*, dihydrochelerythrine, soil-borne diseases, antifungal mechanisms

## Abstract

*Fusarium* species are destructive phytopathogens that cause devastating crop diseases worldwide. The development of botanical pesticides offers a promising strategy for sustainable disease management. This study investigated the antifungal efficacy and mechanism of dihydrochelerythrine (DHC) against *Fusarium oxysporum*. In vitro assays demonstrated that DHC exerted a dose-dependent inhibitory effect by compromising fungal cell membrane integrity, resulting in the leakage of water-soluble carbohydrates and intracellular proteins. Transcriptomic profiling revealed substantial alterations in global gene expression patterns following DHC exposure. Gene Ontology enrichment analysis classified the differentially expressed genes into two principal categories: Biological Process and Molecular Function. Furthermore, KEGG pathway analysis identified 13 significantly up-regulated and 5 down-regulated pathways. Our integrated analysis demonstrates that the antifungal activity of dihydrochelerythrine involves multi-target synergism: it directly disrupts cellular integrity by damaging the cell membrane, while concurrently downregulating key metabolic and signaling pathways, including MAPK signaling, porphyrin metabolism, and mitophagy, thereby impairing stress response and energy homeostasis. These findings identify promising molecular targets—such as ABC transporters and the MAPK pathway.

## 1. Introduction

Fungal and fungus-like pathogens account for over 80% of plant diseases, threatening global food security [1]. *Fusarium* species, which infect over 100 plant species—including maize, wheat, rice, cotton, tomato, banana, eggplant, and tobacco—are among the most important plant pathogens worldwide. They can cause a variety of disease symptoms, such as root or stem rot, canker, vascular wilt, fruit or seed rot, and leaf diseases, often resulting in substantial socioeconomic losses [2]. Recognized for their broad host range, endophytic colonization capacity, and diverse survival and dispersal mechanisms, *Fusarium* pathogens hold major phytopathological significance. *Fusarium oxysporum*, a representative species, is a soil-borne fungus widely distributed across the globe and ranks as the fifth most important plant pathogenic fungus [3]. Through colonization, germination, and dissemination, its spores facilitate the secretion of toxins and effector proteins into plant tissues. This leads to vascular dysfunction, impeding the transport of water and nutrients, and ultimately causing wilting and plant death [4].

Plant secondary metabolites, including alkaloids, phenolics, and terpenes, are recognized for their chemical diversity and potential in eco-friendly disease management [5,6]. However, the development and commercialization of botanical pesticides remain limited. *Chelidonium majus* L., a widely distributed perennial herb of the Papaveraceae family, is a rich source of bioactive alkaloids such as chelidonine, chelerythrine, and sanguinarine, which are known for their diverse pharmacological effects [7,8]. Despite this, the application of *C. majus* extracts or its constituents for plant disease control is underexplored. Our prior research indicating the efficacy of chelerythrine against *Ustilaginoidea virens* and *Xanthomonas oryzae* [9,10] underscores its potential, warranting further investigation into its broader use as a botanical pesticide.

Research on dihydrochelerythrine (DHC), a characteristic alkaloid of *C. majus*, has been largely confined to its medical applications [11], leaving its utility against plant pathogens underexplored. Our previous study provided preliminary evidence of its antifungal properties against *Ustilaginoidea virens* [12]; however, its effects on diverse phytopathogenic fungi and the corresponding mechanisms remain uncharacterized. Building upon our earlier findings, this study was designed to systematically evaluate the inhibitory effect of DHC against *F. oxysporum* and to decipher its mode of action at the molecular level. The objectives are to identify novel molecular targets and to contribute technical insights for the development of DHC-based plant protection strategies.

## 2. Materials and Methods

The *Fusarium oxysporum* strain YFW32, used in this study, was obtained from our previous work [13]. Unless stated otherwise, the fungus was cultured on potato dextrose agar (PDA) plates or in potato dextrose broth (PDB) at 28 °C for 7 days. The experimental design comprised four treatment groups with different DHC concentrations: 0 mg/mL (T0, control), 2.5 × 10^−3^ mg/mL (T2.5), 5.0 × 10^−3^ mg/mL (T5.0), and 7.5 × 10^−3^ mg/mL (T7.5).

The effects of DHC on hyphal morphology were based on hyphal diameter. The spore germination assay was as follows: after 7 d cultivation on PDA, the mycelia were scraped off and the plates were placed on moist gauze for 3 additional days. Spore suspensions were obtained by rinsing plates with DHC solutions (T0, T2.5, T5.0, T7.5) and filtering through four layers of sterile gauze. The suspension was diluted to 50–60 spores per 10 × 40 microscopic field. Then, 80 μL aliquots from each treatment were placed in hemocytometers and incubated at 25 °C in darkness for 10 h. The rate of spore germination was calculated.

To determine pathogen cell membrane permeability, mycelia of *F. oxysporum* pre-cultured on PDA for 7 days were harvested and resuspended in 0.01 M phosphate-buffered saline (PBS). The suspensions were then treated with DHC to achieve final concentrations of 0 (T0, control), 2.5 × 10^−3^ (T2.5), 5.0 × 10^−3^ (T5.0), and 7.5 × 10^−3^ (T7.5) mg/mL. Following treatment, the mixtures were incubated at 28 °C and sampled at 30, 60, 90, 120, and 150 min. At each time point, the suspensions were centrifuged at 6400× *g* for 5 min, and the absorbance of the resulting supernatant was measured at 280 nm to assess the relative content of released cellular materials [12].

To determine water-soluble carbohydrates (WSC) in culture medium after DHC application, sterilized PDB was supplemented with DHC solution to prepare drug-containing media. Each flask was inoculated with five 8 mm mycelial plugs obtained from a 7-day-old culture of *F. oxysporum* and incubated at 180 rpm. Culture samples (1 mL) were collected at specified time intervals (0, 6, 12, 24, 48, and 96 h). Subsequently, the samples were centrifuged at 6400× *g* for 5 min. The WSC content in the resulting supernatant was quantified using the anthrone method [14], with trehalose as a positive control for WSC determination. The relative change in WSC content for each DHC treatment was calculated against the respective T0 (control) baseline.

To determine the effects of DHC on intracellular protein, following a 7-day liquid shake-flask culture, *F. oxysporum* was subjected to corresponding concentrations of DHC and incubated for an additional 2 days. The mycelia were then harvested by centrifugation (6400× *g*, 4 °C, 5 min), washed thoroughly with sterile water, and gently blotted dry. Subsequently, 0.5 g of mycelia from each treatment was homogenized on ice in 2.5 mL of 0.05 mol/L Tris extraction buffer. The resulting homogenate was centrifuged (6400× *g*, 4 °C, 10 min) to obtain a clear supernatant. For protein quantification, 1 mL of the supernatant was mixed with 5 mL of 0.01% Coomassie Brilliant Blue G-250 solution, and the protein concentration was determined according to the Bradford method [15], with BSA (fraction V, BOSF, Hefei, China) as a positive control for intracellular protein measurement.

For RNA-seq library preparation and sequencing, *F. oxysporum* was cultured in PDB for 3 days as described previously. The cultures were then treated with DHC at final concentrations of 0 (T0), 2.5 × 10^−3^ (T2.5), 5.0 × 10^−3^ (T5.0), and 7.5 × 10^−3^ (T7.5) mg/mL and incubated for another 3 days. Subsequently, 2 mL of each fungal suspension was collected with RNase-free tips, and the mycelia were pelleted by centrifugation at 10,000× *g* for 2 min at 4 °C. After discarding the supernatant, the cell pellets were labeled, flash-frozen in liquid nitrogen for 30 min, and stored at −80 °C until RNA extraction. Total RNA was extracted using a Column System Total RNA Extraction and Purification Kit (Sangon Biotech, Shanghai, China) following the manufacturer’s protocol. RNA quality was assessed by measuring concentration and purity with a Nanodrop ND-2000 spectrophotometer (NanoDrop, Wilmington, DE, USA) and confirming integrity via 1% agarose gel electrophoresis. The RNA-seq libraries were constructed and pair-end sequenced (2 × 150 bp) by MajorBio (Shanghai, China) using standard Illumina protocols, and the resulting datasets were analyzed on the Illumina NovaSeq X Plus platform (Illumina San Diego, CA, USA).

For bioinformatic analysis, raw sequencing reads were first processed for quality control on the Majorbio Cloud Platform (https://cloud.majorbio.com/). The resulting high-quality clean reads were then aligned to the *F. oxysporum* reference genome (FO2) using RSEM software (Version 1.3.3) to quantify gene expression levels. Differential expression analysis between the T5.0 and T0 groups was carried out with the DESeq2 package [16], applying a threshold of an adjusted *p*-value (FDR) < 0.05 and an absolute log_2_ fold change >1 to identify significantly differentially expressed genes (DEGs). To functionally characterize these DEGs, Gene Ontology (GO) enrichment analysis was performed using Goatools, and Kyoto Encyclopedia of Genes and Genomes (KEGG) pathway enrichment analysis was conducted with the Python scipy package (Version 1.0.0) [17]. Fisher’s exact test was employed for both analyses, and terms or pathways with a *p*-value < 0.05 were deemed significantly enriched.

Data are from three biological replicates unless otherwise noted. Statistical analysis was performed using SPSS version 22 (SPSS Inc., Chicago, IL, USA). One-way analysis of variance (ANOVA) was conducted to assess the data. Subsequently, Dunnett’s multiple comparison test was employed to determine significant differences, with *p* < 0.05 considered statistically significant.

## 3. Results

### 3.1. Effects of DHC on Hyphal Morphology and Spore Germination

**Figure 1:**
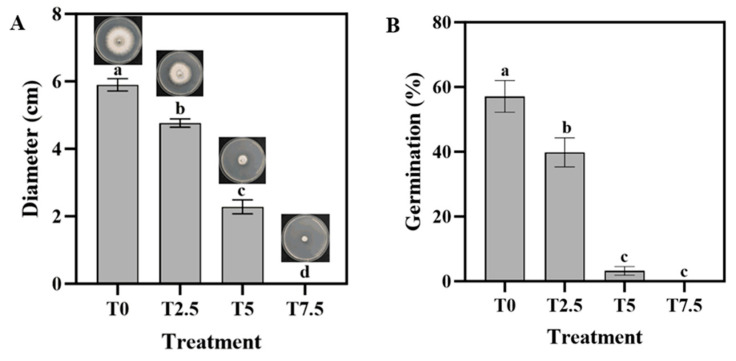
Effects of DHC on hyphal morphology and spore germination of *F. oxysporum.* (**A**) Mycelial diameter and morphology of *F. oxysporum* on PDA plates treated with different concentrations of DHC. (**B**) Spore germination. Data are presented as mean ± standard deviation (SD) (n = 3), with significant differences indicated by different lowercase letters (*p* < 0.05, one-way ANOVA followed by Duncan’s multiple range test, analyzed using SPSS version 22).

As shown in the PDA media, the mycelial diameter progressively decreased with increasing DHC concentrations (Figure 1A). No mycelial growth was observed in the T7.5 treatment (7.5 × 10^−3^ mg/mL). Spore germination assays also demonstrated that spores subjected to T7.5 treatment failed to germinate.

### 3.2. Effects of DHC on Cell Membrane Permeability

**Figure 2:**
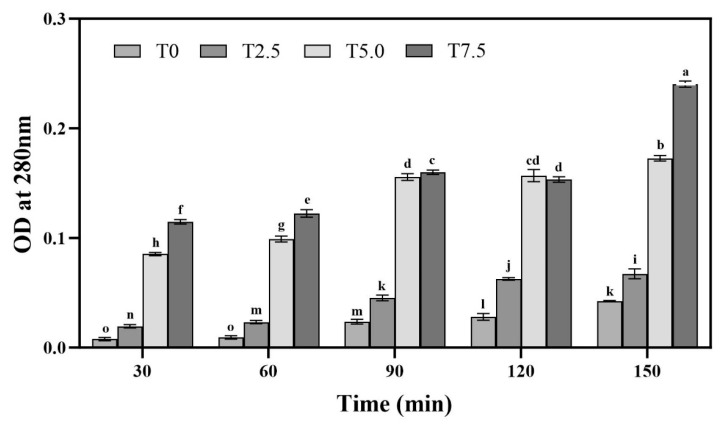
Effects of DHC on cell membrane permeability of *F. oxysporum*. Data are presented as mean ± standard deviation (SD) (n = 3), with significant differences indicated by different lowercase letters (*p* < 0.05, one-way ANOVA followed by Duncan’s multiple range test, analyzed using SPSS version 22).

Analysis of the absorbance curves revealed a significant time-dependent increase (*p* < 0.05) across all treatments, consistent with progressive hyphal cell rupture during incubation in buffer (Figure 2). Furthermore, at each time point within the 90 min period, the absorbance values correlated directly with the DHC concentration. At 90 min, the optical density at 5.0 × 10^−3^ mg/mL (T5.0) was 2.43-fold higher (*p* < 0.05) than that of T2.5 (2.5 × 10^−3^ mg/mL). This positive correlation indicates that higher DHC concentrations induced greater mycelial permeability, thereby resulting in an elevated release of intracellular proteins into the solution.

### 3.3. Effects of DHC on Water-Soluble Carbohydrates in the Culture

**Figure 3:**
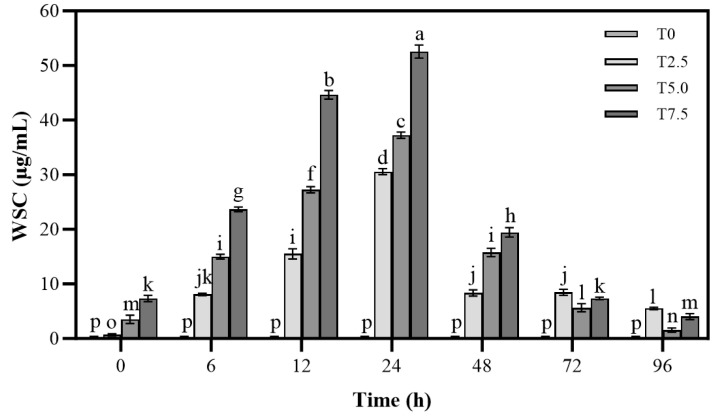
Effects of DHC on water-soluble carbohydrate of *F. oxysporum*. Data are presented as mean ± standard deviation (SD) (n = 3), with significant differences indicated by different lowercase letters (*p* < 0.05, one-way ANOVA followed by Duncan’s multiple range test, analyzed using SPSS version 22).

As shown in Figure 3, the changes in water-soluble carbohydrate in culture medium treated with DHC were evaluated in this study. The WSC level increased continuously with prolonged incubation time. Within 24 h, higher DHC concentrations led to greater WSC accumulation, peaking at 24 h (52.54 µg/mL of T7.5 treatment), followed by a subsequent decline.

### 3.4. Effects of DHC on Intracellular Protein

**Figure 4:**
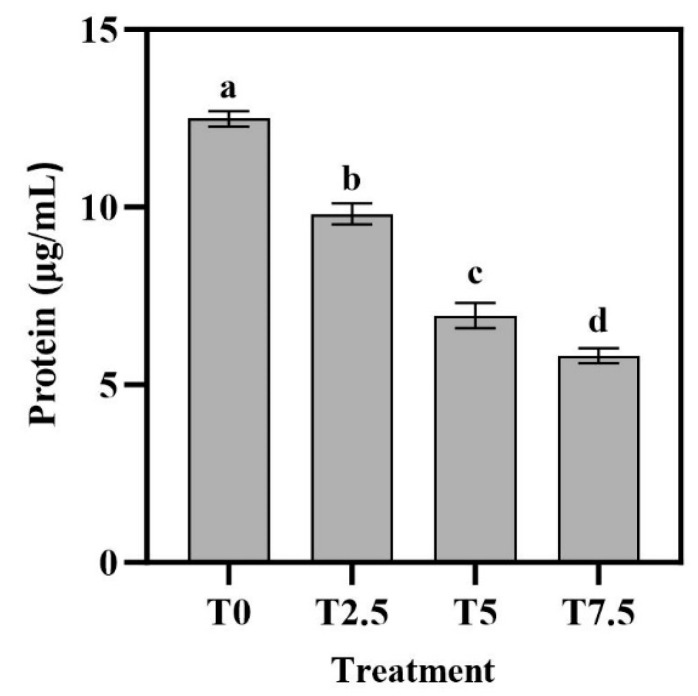
Effects of DHC on intracellular proteins of *F. oxysporum*. Data are presented as mean ± standard deviation (SD) (n = 3), with significant differences indicated by different lowercase letters (*p* < 0.05, one-way ANOVA followed by Duncan’s multiple range test, analyzed using SPSS version 22).

As shown in Figure 4, DHC treatment significantly suppressed fungal protein synthesis in a concentration-dependent manner. The highest value (12.49 µg/mL) occurred in the T0 treatment (*p* < 0.05). The lowest value was 5.82 µg/mL in T7.5 treatment (*p* < 0.05). The results clearly show that the total protein content in *Fusarium oxysporum* decreased progressively with increasing DHC concentrations.

### 3.5. Differential Expression Analysis

**Figure 5:**
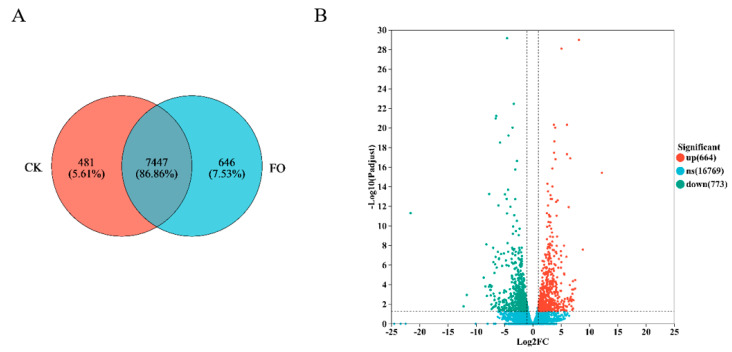
Changes in gene expression of *F. oxysporum* after DHC application. (**A**) Venn diagram showing the overlap of expressed genes in CK (control group) and FO (treatment group); (**B**) Volcano plot of DEGs. The *x*-axis represents log_2_ fold change (log_2_FC) in gene expression (FO vs. CK), and the *y*-axis represents −log_10_ (adjusted *p*-value, FDR).

Transcriptome sequencing was performed on *Fusarium oxysporum* samples treated with 5.0 × 10^3^ mg/L (T5.0) and no addition (T0) groups. The Venn diagram (Figure 5A) revealed 7447 common genes between the two treatments, with 481 and 646 genes unique to CK and T5.0, respectively. Differentially expressed genes (DEGs) were screened, and as shown in Figure 5B, compared to CK, the DHC treatment resulted in 664 up-regulated and 773 down-regulated genes.

### 3.6. GO Enrichment Analysis of Differentially Expressed Genes

**Figure 6:**
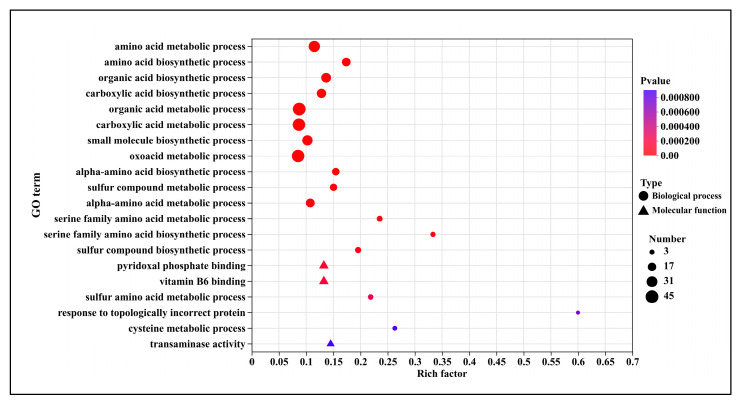
GO Enrichment analysis of up-regulated genes after DHC application.

GO enrichment analysis of differentially expressed genes (DEGs) revealed their distribution across two primary categories: Biological Process and Molecular Function. Compared to T0, the T5.0 treatment exhibited enrichment in 142 up-regulated GO terms. As illustrated in Figure 6, the top 20 significantly up-regulated terms were primarily associated with molecular functions such as pyridoxal phosphate binding (13 genes), vitamin B6 binding (13 genes), and transaminase activity (9 genes), as well as biological processes including amino acid metabolism (35 genes) and the biosynthesis of organic and carboxylic acids (50 genes). Based on the bubble plot analysis, the most significantly up-regulated term was “response to topologically incorrect protein” (3 genes), followed by “serine family amino acid biosynthetic process” (6 genes).

**Figure 7:**
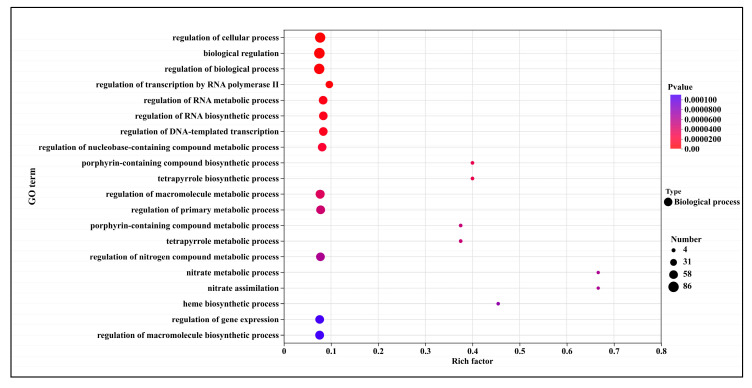
GO Enrichment analysis of down-regulated genes after DHC application.

The GO enrichment analysis of down-regulated differentially expressed genes is presented in Figure 7. In T5.0, the top 20 significantly up-regulated terms were predominantly within the biological process category. These terms encompassed the regulation of intracellular processes (82 genes), biological regulation (86 genes), regulation of biological processes (82 genes), regulation of RNA synthesis and metabolism (55 genes), the tetrapyrrole biosynthetic process (6 genes), and macromolecule metabolic regulation (62 genes). The most significantly altered processes were nitrate metabolic process (4 genes), nitrate assimilation (4 genes), and heme biosynthetic process (5 genes).

### 3.7. KEGG Pathway Annotation of Differentially Expressed Genes

**Figure 8:**
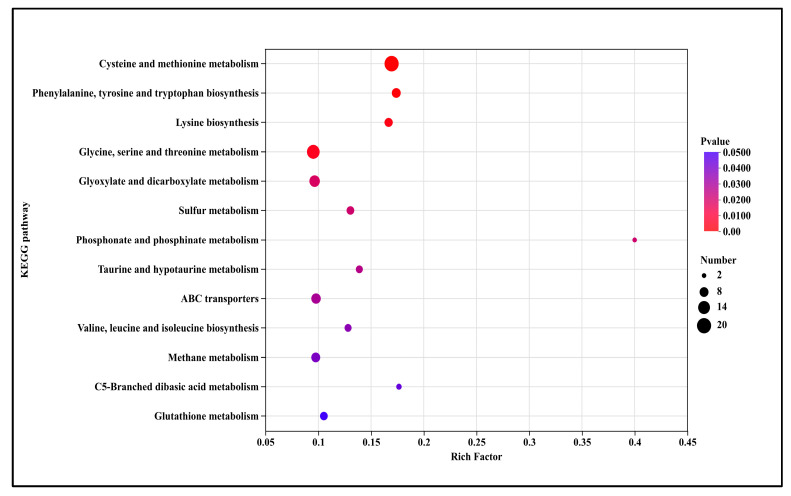
KEGG pathway annotation of up-regulated genes after DHC treatment.

The bubble plot displays the significantly enriched 13 up-regulated KEGG pathways in T5.0 treatment (Figure 8). These included amino acid and nitrogen metabolism (cysteine and methionine metabolism (3.6-fold), phenylalanine, tyrosine and tryptophan biosynthesis (3.7-fold), lysine biosynthesis (3.6-fold), glycine, serine and threonine metabolism, valine, leucine and isoleucine biosynthesis (2.8-fold)), energy and carbon metabolism (glyoxylate and dicarboxylate metabolism (2.1-fold), methane metabolism (2.1-fold), C5-branched dibasic acid metabolism (3.8-fold)), sulfur, phosphorus and specialized molecule metabolism (sulfur metabolism (2.8-fold), phosphonate and phosphinate metabolism (8.5-fold), taurine and hypotaurine metabolism (3.0-fold), glutathione metabolism (2.2-fold)) and transport system (ABC transporters (2.1-fold)).

**Figure 9:**
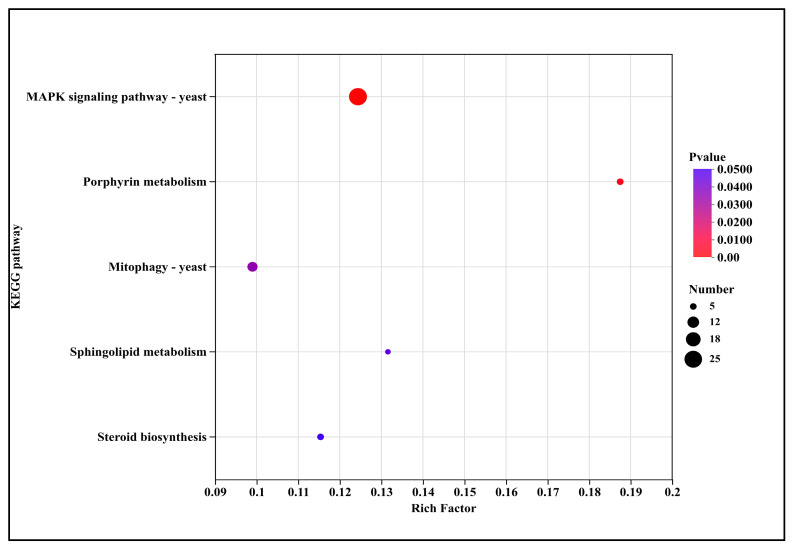
KEGG pathway annotation of down-regulated genes after DHC treatment.

The significantly down-regulated five pathways (Figure 9) encompassed signal transduction (MAPK signaling pathway—yeast (2.5-fold)), metabolic pathways including porphyrin metabolism (3.7-fold), sphingolipid metabolism (2.6-fold), and steroid biosynthesis (2.3-fold), as well as cellular processes such as mitophagy—yeast (2.0-fold).

## 4. Discussion

Dihydrochelerythrine (DHC) has been demonstrated to exhibit significant inhibitory activity against *Ustilaginoidea virens*. Its antifungal mechanism involves the induction of mitochondrial dysfunction and cell apoptosis [12]. In this study, using *F. oxysporum* as the target organism, we further investigated the antifungal action of DHC from four perspectives: pathogen growth, cell membrane permeability, intracellular protein synthesis, and transcriptomic differential expression.

Compared to CK, cultures treated with DHC exhibited a concentration-dependent reduction in hyphal diameter and a graded decrease in spore germination rate (Figure 1). Transcriptomic analysis revealed that the reduction in hyphal diameter was primarily attributable to the significant downregulation of key genes involved in the cell wall biosynthesis pathway. These include chitin synthase *CHSV* (e.g., *FOXG_04162*) and glucan synthase *FKS1* (e.g., *FOXG_03721*), whose impaired expression likely hindered cell wall assembly and consequently slowed the growth rate [16,17]. Concurrently, aberrant expression of genes in the energy metabolism pathway may have exacerbated this phenotype [18], as evidenced by the significant downregulation of an oxidative phosphorylation (ATP synthase) gene (e.g., *FOXG_00773*). The inhibition of spore germination is likely due to the suppressed expression of *FOSTUA* (e.g., *FOXG_05278*), a core transcription factor governing conidiation, thereby disrupting the normal sporulation developmental program [19].

The cell membrane is a critical structure for maintaining osmotic equilibrium, material exchange, and signal transduction in fungal cells [20]. Compromising its integrity represents the initial step in the action of many antifungal agents. In this study, cell membrane permeability assays revealed that the absorbance of the mycelial suspension in buffer increased over time (*p* < 0.05), regardless of DHC treatment, indicating a certain degree of natural rupture of mycelia under buffer conditions. At each time point within 90 min, the high-concentration DHC treatment showed significantly higher absorbance (Figure 2), reflecting increased protein content in the solution and markedly enhanced mycelial permeability. This finding is consistent with previous reports that DHC causes deformities in the cell wall, nuclear membrane damage, and concentration-dependent leakage of intracellular contents in *U. virens* spores [12]. These results suggest that DHC may disrupt the physical structure of the fungal cell membrane (e.g., integrity of the phospholipid bilayer), leading to the leakage of macromolecules such as intracellular proteins, thereby interfering with normal cellular metabolism [21]. The downregulation of membrane function-related genes (*FOXG_03480*, *FOXG_04752*, *FOXG_11113*, etc.) in the transcriptome results supports this finding.

This study found that the WSC content in the culture medium of DHC-treated groups increased continuously over time, with significantly higher (*p* < 0.05) accumulation in the high-concentration DHC group (Figure 3). This phenomenon can be explained from two perspectives: On the one hand, DHC compromises cell membrane integrity, leading to passive leakage of intracellularly stored WSCs into the medium. Similarly, chelerythrine (CHE, a structural analog of DHC) has been observed to induce leakage of intracellular nutrients in *U. virens*, an effect attributed to the binding of alkaloid molecules to membrane phospholipids, which alters membrane fluidity and transporter activity [22]. Furthermore, the inhibitory effect of DHC on protein synthesis in *F. oxysporum* corroborates its disruption of cellular metabolism: as the DHC concentration increased, fungal protein content decreased significantly, showing a concentration-dependent suppression (Figure 4). This aligns with reports that CHE inhibits hyphal protein synthesis in *U. virens* [9]. It is hypothesized that DHC may impair ribosomal structure or inhibit post-transcriptional translation processes, thereby reducing the production of functional proteins and ultimately leading to metabolic dysfunction. This is further supported by the transcriptomic data, which showed decreased expression of both ribosomal function genes (*ENSRNA049509142*, *ENSRNA049510778*, *FOXG_04177*, etc.) and genes involved in post-transcriptional translation processes (*FOXG_04148*, *FOXG_13829*, *FOXG_02368*, etc.).

To elucidate the mechanism of action of DHC at the molecular level, transcriptome sequencing was performed on *F. oxysporum* treated with 5.0 × 10^3^ mg/L DHC (T5.0) and a control group (T0). DHC treatment resulted in the up-regulation of 664 genes and the down-regulation of 773 genes (Figure 5). This differential expression pattern resembles the impact of DHC on the proteome of *U. virens*—a previous TMT-based quantitative proteomic study identified 311 differentially expressed proteins (DEPs) in *U. virens* upon DHC treatment, among which 132 were up-regulated and 179 down-regulated, with DEPs predominantly enriched in metabolic pathways [12]. These findings suggest that the regulatory effect of DHC on gene/protein expression may be conserved across different pathogenic fungi.

GO enrichment analysis indicated that up-regulated DEGs following DHC treatment were enriched in molecular functions—such as pyridoxal phosphate binding, vitamin B6 binding, and transaminase activity—as well as biological processes including amino acid metabolism and organic acid biosynthesis. Notably, “response to topologically incorrect proteins” and “serine family amino acid biosynthetic process” were significantly up-regulated (Figure 6). The accumulation of misfolded proteins suggests possible disruption of endoplasmic reticulum function or chaperone inhibition by DHC. Concurrent up-regulation of amino acid metabolism may represent a compensatory response to protein leakage or synthesis inhibition [23]. These findings align with reported enrichment of metabolic processes in CHE-treated *U. virens* [9], supporting a common mechanism by which alkaloids perturb fungal metabolic homeostasis.

Down-regulated DEGs were primarily enriched in biological processes including “nitrate metabolism”, “nitrate assimilation”, and “heme biosynthesis”, as well as regulatory pathways such as RNA anabolic regulation and macromolecule metabolic regulation. The suppression of nitrate assimilation—a key pathway for nitrogen acquisition—likely impairs the fungal ability to synthesize proteins and nucleic acids, consistent with the observed inhibition of protein synthesis in this study (Figure 4). Concurrently, the down-regulation of heme biosynthesis may disrupt the function of cytochromes and peroxidases, affecting mitochondrial electron transport and oxidative stress response [24]. Similarly, DHC treatment in *U. virens* led to reduced expression of cytochrome c oxidase subunits (e.g., COX5B, COX6), resulting in decreased mitochondrial membrane potential and ROS accumulation [12]. These findings suggest that DHC-mediated inhibition of heme metabolism and mitochondrial function may represent a conserved mechanism across fungal species.

KEGG pathway enrichment analysis further elucidated the core pathways modulated by DHC in *F. oxysporum*. Among up-regulated pathways, amino acid and nitrogen metabolism (e.g., cysteine-methionine metabolism, phenylalanine-tyrosine-tryptophan biosynthesis), energy and carbon metabolism (e.g., glyoxylate-dicarboxylate metabolism), and ABC transporter pathways were most prominent (Figure 7). The up-regulation of ABC transporters—key fungal efflux pump systems—likely represents a resistance response to DHC, enhancing its extrusion and reducing intracellular accumulation [25,26]. Concurrent up-regulation of amino acid metabolic pathways may facilitate repair of DHC-induced protein damage or help maintain osmotic homeostasis, corroborating the GO enrichment results related to amino acid biosynthesis (Figure 6). Additionally, up-regulation of glutathione metabolism is noteworthy. As glutathione serves as a critical intracellular antioxidant, its enhanced metabolism may function to scavenge DHC-induced ROS. This complements previous observations of ROS accumulation in DHC-treated *U. virens* [12], collectively reflecting a conserved oxidative stress response mechanism to DHC across fungal species. Transcriptomic data presented here were obtained from samples treated with DHC for 3 days. While these results clearly indicate “the occurrence of a response”, the transcriptome data alone cannot determine whether this up-regulation is sustained long-term. Future functional validation experiments—such as drug susceptibility assays, reversibility tests, functional knockdown/knockout studies, and genetic mutation analysis—are essential to precisely delineate the underlying mechanisms and their persistence. This clarification is crucial for accurately assessing the therapeutic potential of the compound and for guiding the selection of optimal application strategies.

Down-regulated pathways were primarily enriched in signal transduction (e.g., yeast MAPK signaling pathway), metabolism (e.g., porphyrin and sphingolipid metabolism), and cellular processes (e.g., mitophagy in yeast) (Figure 8). Suppression of the MAPK pathway, which centrally regulates fungal growth, differentiation, and stress response, likely compromises the ability to perceive and adapt to external stress, thereby inhibiting hyphal growth and spore germination [27,28]. The marked down-regulation of porphyrin metabolism aligns with the inhibition of “heme biosynthesis” observed in GO analysis, further supporting that DHC impairs mitochondrial function and oxidative metabolism by disrupting heme synthesis (Figure 9). Additionally, the down-regulation of mitophagy suggests a failure to clear damaged mitochondria, leading to their intracellular accumulation and exacerbating apoptosis [29]. This significantly differs from the pathway of CHE in *U. virens*, where down-regulation of SNARE-mediated vesicle trafficking and oxidative phosphorylation disrupted endomembrane function and energy metabolism [9]. These results are particularly important for controlling diseases caused by *Fusarium*, which causes widespread damage.

Our results demonstrate that the antifungal activity of DHC against *F. oxysporum* involves multi-target and multi-pathway synergism. On one hand, DHC compromises cell membrane integrity, leading to leakage of proteins and water-soluble carbohydrates, thereby directly disrupting cellular metabolism. On the other hand, it downregulates key pathways including MAPK signaling, porphyrin metabolism, and mitophagy, impairing stress response and energy production. Although compensatory up-regulation of amino acid metabolism and ABC transporters occurs, cumulative damage ultimately leads to cellular dysfunction. The results also identify promising targets—such as ABC transporters and MAPK pathway genes—for controlling *Fusarium* wilt and other soil-borne diseases.

Here, we primarily focused on the inhibitory effect and mechanism of DHC against *F. oxysporum* based on in vitro experiments, without evaluating its control efficacy through plant cultivation trials. It is imperative to conduct in planta assays to evaluate DHC’s efficacy under natural infection conditions and its impact on soil microbiota. It is also essential to assess possible phytotoxic effects and determine an optimal field application dosage. In addition, we tested the stability of DHC in the broth containing DHC and observed no significant change. However, its stability under field conditions requires further investigation, as this is a critical indicator of its practical application potential. Moreover, given the numerous compounds present in *Chelidonium majus*, it remains unclear whether they may produce synergistic or antagonistic effects with DHC. Further research could begin by comparing the combined effects of DHC with those of the individual compounds, such as chelerythrine, to investigate potential synergistic or antagonistic interactions. This would contribute to the development of more effective bio-based composite pesticides, thereby addressing potential resistance issues associated with single-compound applications. Such investigations would provide more comprehensive theoretical support for the industrial application of DHC and promote the utilization of botanical fungicides in the management of soil-borne diseases.

## Data Availability

The original data presented in the study are openly available in GenBank under BioProject ID PRJNA1348785.
